# Transformer fault diagnosis method based on TLR-ADASYN balanced dataset

**DOI:** 10.1038/s41598-023-49901-9

**Published:** 2023-12-27

**Authors:** Shan Guan, Haiqi Yang, Tongyu Wu

**Affiliations:** School of Mechanic Engineering, Northeast Electric Power University, Jilin, 132012 China

**Keywords:** Electrical and electronic engineering, Power distribution

## Abstract

As the cornerstone of transmission and distribution equipment, power transformer plays a very important role in ensuring the safe operation of power system. At present, the technology of dissolved gas analysis (DGA) has been widely used in fault diagnosis of oil-immersed transformer. However, in the actual scene, the limited number of transformer fault samples and the uneven distribution of different fault types often lead to low overall fault detection accuracy or a few types of fault misjudgment. Therefore, a transformer fault diagnosis method based on TLR-ADASYN balanced data set is presented. This method effectively addresses the issue of samples imbalance, reducing the impact on misjudgment caused by a few samples. It delves deeply into the correlation between the ratio of dissolved gas content in oil and fault type, eliminating redundant informations and reducing characteristic dimensions. The diagnostic model SO-RF (Snake Optimization-Random Forest) is established, achieving a diagnostic accuracy rate of 97.06%. This enables online diagnosis of transformers. Comparative analyses using different sampling methods, various features, and diverse diagnostic models were conducted to validate the effectiveness of the proposed method. In conclusion, validation was conducted using a public dataset, and the results demonstrate that the proposed method in this paper exhibits strong generalization capabilities.

## Introduction

As a hub in electrical power systems, transformers directly influence the stability and reliability of power system operations. Therefore, accurately understanding the health status of transformers is of paramount importance for ensuring the safe and stable operation of the power system. When transformers experience insulation aging, gases such as H_2_, CH_4_, C_2_H_6_, C_2_H_4_, C_2_H_2_, CO, and CO_2_ dissolve in the insulating oil. The composition and concentration of dissolved gases can reflect the current operational status of the transformer^1^. Common analysis methods include the IEC three-ratio method^2^, Rogers’ ratio method^3^, and the Duval method^4^. Recent studies have optimized the coding of the three-ratio diagnosis using dissolved gases, further exploring transformer diagnostics^5^. Additionally, a method based on fuzzy three-ratio and case matching for transformer fault diagnosis has been proposed, using the Euclidean distance method to calculate the similarity between target cases and cases in the selected subspace, with the method being validated through practical examples^6^. However, these methods, while operationally straightforward, lack depth in characterizing fault features and have limitations, with fuzzy and unclear coding boundaries, leading to lower fault recognition accuracy^7^. Scholars have proposed the use of non-coded ratio methods^8,9^ after conducting extensive comparative experiments and literature reviews. These methods only require gas concentration ratios and utilize coding methods based on the percentage of key gases in total gases or total hydrocarbon concentrations to reflect the relationship between features and fault types. In Ref.^10^, by combining the non-coded ratio method with deep dense neural networks, a model’s judgment and generalization capabilities have been improved. In Ref.^11^, causing the non-coded ratio method, nine-dimensional fault features were extracted and directly input into the XGBoost diagnostic model, achieving a diagnostic accuracy of 92.7%. However, during the diagnosis process, as the dimensionality of features increases, redundant information also increases, leading to an increase in the computational complexity of the model. Thus, eliminating redundant information, reducing model computation time, and enhancing diagnostic accuracy are among the key focuses of this research.

With the development of machine learning theory, models such as Support Vector Machines^12,13^ (SVM), Convolutional Neural Networks^14–16^ (CNN), Extreme Learning Machines^17^ (ELM), Long Short-Term Memory Networks^18,19^ (LSTM) and U-Net^20^ have been effectively applied in classification and recognition. Yet, these methods require a large number of training samples, and in practical transformer operation, the fault rate is low, with varying frequencies of different fault types. It is challenging to meet the training requirements for artificial intelligence diagnostics with imbalanced small samples. Currently, experts and scholars have conducted extensive research to address the imbalance in datasets, proposing solutions from both the sample and algorithm perspectives. Sample-based solutions include oversampling and undersampling methods. Undersampling achieves sample balance by removing some majority class samples but is prone to eliminating valuable information and is not widely adopted^21^. Oversampling, on the other hand, balances the dataset by generating minority class samples^22–24^. Algorithm-based solutions primarily include ensemble learning^25^ and cost-sensitive methods^26^. The ADASYN algorithm was used to augment minority class samples in a study, further enhancing equipment fault classification performance^27^. Another study proposed enhancing sample intra-class feature aggregation by increasing the number of clusters based on imbalance degree and K-means clustering^28^. This improved sample identifiability. Although these methods have reduced the occurrence of misclassification and omission of minority class samples to some extent, they do not consider boundary samples and noise when synthesizing new samples, resulting in the problem of fuzzy classification boundaries.

To address these issues, this paper tackles the problem of recognizing and classifying imbalanced small sample data from both the sample and algorithm levels, proposing a transformer fault diagnosis method based on a TLR-ADASYN balanced dataset. Firstly, the influence of noise and boundary samples is eliminated before balancing the data. Secondly, to address the limitations of traditional diagnostic methods in characterizing complex internal fault features of transformers, multi-dimensional ratio features are constructed. These features delve deeper into the correlation between the ratios of dissolved gas contents in the oil and the state types, eliminating the impact of redundant information and improving operational efficiency. Finally, a transformer fault diagnosis model is established, and the effectiveness of the proposed method is validated through real-world data.

## Synthetic oversampling of boundary samples based on Tomek link

### ADASYN minority-class sample synthesis technique

ADASYN is an adaptive data synthesis method proposed by He et al.^29^. The method adaptively synthesizes different numbers of new samples according to the distribution of minority samples. The specific algorithm steps are as follows.

Suppose the training set is ${\text{D}}$, which contains ${\text{m}}$ samples, $\left\{ {x_{i} ,y_{i} } \right\},i = 1,2, \ldots ,m$, $x_{i}$ is represented as a sample of the feature space ${\text{X}}$, $y_{i} \in {\text{Y}} = \left\{ { - 1,1} \right\}$. ${\text{m}}_{s}$ and ${\text{m}}_{l}$ represent the number of minority samples and majority samples, respectively. Hence, ${\text{m}}_{s} \le {\text{m}}_{l}$ and ${\text{m}}_{s} + {\text{m}}_{l} = m$ exist.

Calculate class unbalance degree:

1 $$d = \frac{{m_{s} }}{{m_{l} }},$$

where $$d \in \left( {0,1} \right]$$.

Calculate the total number of samples of a few classes that need to be synthesized ${\text{G}}$:

2 $$G = \left( {m_{l} - m_{s} } \right) \times \beta ,$$

where $\beta \in \left[ {0,1} \right]$ is the random number of the interval, representing the unbalance degree after the generation of new data. $\beta = 1$ indicates that the positive/negative ratio after sampling is 1:1.

Calculate the proportion of majority classes in K-nearest neighbors:

3 $$r_{i} = \frac{{\Delta_{i} }}{K}.$$

According to the sample weight, calculate the number of new samples that need to be generated for each minority sample.

4 $$g = G \times \hat{r}_{i} .$$

To calculate the number of samples generated for each minority sample according to ${\text{g}}$:

5 $$S_{i} = \left( {x_{iz} - x_{i} } \right) \times \lambda ,$$

where $S_{i}$ is the synthesized new sample, $X_{i}$ is the i-th sample in the minority sample, $\left( {x_{iz} - x_{i} } \right)$ is the m-dimensional vector representing the difference between the two minority samples, and $\lambda$ is the random number in the $\left[ {0,1} \right]$ interval.

### TLR-ADASYN equilibrium dataset

Tomek^30^ improved the convolutional neural network in 1976 and proposed a new framework, which undersampled the boundary samples without destroying the potential information. Two adjacent samples of different classes can be connected into a Tomek Link. Its formation process is as follows:

Suppose there are two types of sample sets $C_{1}$ and $C_{2}$, and the corresponding samples are $u_{i} \left( {i \in \left\{ {l, \ldots ,n} \right\}} \right)$ and $v_{i} \left( {i \in \left\{ {l, \ldots ,m} \right\}} \right)$ respectively. Define distance $dist\left( {u_{i} ,v_{i} } \right) = \left\| {u_{i} - v_{i} } \right\|$, If there are no other samples $v_{p}$ or $u_{q}$ that satisfy the conditions of $dist\left( {u_{q} ,v_{j} } \right) < dist\left( {u_{i} ,v_{j} } \right)$ or $dist\left( {u_{q} ,v_{j} } \right) < dist\left( {u_{i} ,v_{j} } \right)$. Thus, $\left( {u_{i} ,v_{j} } \right)$ can form a pair of Tomek chain.

For each $u_{i} \in C_{1}$, find the nearest $v_{p} \in C_{2}$, form a chain $l_{12}$ set and save it.

6 $$l_{12} = \left\{ {\left( {\mu_{i} - v_{p} } \right)\left| {\mu_{i} \in C_{1} } \right.} \right\}.$$

For each $v_{j} \in C_{2}$, find the nearest $C_{2}$, form a chain $l_{12}$ set and save it.

7 $$l_{21} = \left\{ {\left( {\mu_{q} - v_{j} } \right)\left| {v_{j} \in C_{2} } \right.} \right\}.$$

$l_{12}$ and $l_{21}$ constitute Tomek link $\Pi$:

8 $$\prod = l_{12} \cap l_{21} .$$

Tomek Link reduces noise and boundary data by eliminating problematic pairs. To prevent the classifier from favoring the majority class too much, ADASYN expands the minority class data, addressing the bias issue.

## Transformer fault diagnosis model based on SO-RF

### Random forest

RF^31^ belongs to one of the integrated algorithms and it is a set $\left\{ {{\text{h}}\left( {X,\theta_{k} } \right),k = 1,2, \ldots ,n} \right\}$ composed of k decision tree classification models, the set is extracted by Booststrap sampling method, and the final classification result is obtained by subtree voting. The steps to build an RF classification model are as follows.

*Step 1* Using Booststrap sampling, samples with the same capacity are drawn from the training set N to generate the training subset.

*Step 2* It is assumed that the training subset has S features, and s samples selected at random are taken as the split feature subset and split by CART algorithm.

*Step 3* Repeat Step1 to Step2 for n times to generate subtree and build RF model.

*Step 4* Test sets are used to verify the reliability of RF models, and the final classification results are decided by voting.

### Snake optimization algorithm

Snake Optimization algorithm^32^ is a new meta-heuristic algorithm proposed in 2022, which mainly simulates the foraging and reproduction behavior of snakes. The algorithm has the advantages of simple principle and good optimization performance. The specific principle is as follows.

#### Initialize

Snake population initialization is shown in Eq. (9):

9 $$X_{i} = \, X_{\min } + \, r \times \left( {X_{\max } - X_{\min } } \right),$$

where $X_{i}$ is the position of the i-th snake; r is a random number in the range [0,1]; $X_{\max }$ and $X_{\min }$ are the upper and lower boundaries.

#### The population was divided into two groups, male and female, and Temp and Q were defined

Suppose the number of males is 50% and the number of females is 50%. The population is divided into two groups: male and female. Define the temperature Temp and the amount of food Q, and find the best individual in each group. Temp and Q can be expressed by formulas (10) and (11):

10 $$Temp = \exp \left( {\frac{ - t}{T}} \right),$$

11 $$Q = c_{1} \times \exp \left( {\frac{t - T}{T}} \right),$$

where *t* represents the current number of iterations; *T* is the maximum number of iterations; $c_{1}$ is a constant, usually 0.5.

#### Exploration phase

If $Q < Threschold\left( {0.25} \right)$, the snake randomly selects a location to search for food and updates the location. The exploration phase is shown in Eq. (12):

12 $$X_{i,m} \left( {t + 1} \right) = X_{rand,m} \left( t \right) \pm c_{2} \times A_{m} \times \left( {\left( {X_{\max } - X_{\min } } \right) \times rand + X_{\min } } \right),$$

where $X_{i,m}$ is the male position; $X_{rand,m}$ is the location of the randomly selected male; $rand$ is the random number of [0,1]; $c_{2}$ is a constant, usually 0.05; $A_{m}$ The ability to find food for males.

#### Development phase

Under conditions $Q > Threschold$ is satisfied, if $Q > Threschold\left( {0.6} \right)$, the snakes are in a hot state and looking for food, the position is updated as shown in Eq. (13):

13 $$X_{i,j} \left( {t + 1} \right) = X_{food} \left( t \right) \pm c_{3} \times Temp \times rand \times \left( {X_{food} - X_{i,j} \left( t \right)} \right),$$

where $X_{i,f}$ is the position of the snake individual; $X_{food}$ is the optimal position of individual snake. $rand$ is the random number of [0,1]; $c_{3}$ is a constant, usually 2.

If $Q < Threschold\left( {0.6} \right)$, the temperature is cold, the snake will be in fight mode or mating mode.

① Combat pattern

14 $$X_{i,m} \left( {t + 1} \right) = X_{i,m} \left( t \right) + c_{3} \times FM \times rand \times \left( {Q \times X_{best,f} - X_{i,m} \left( t \right)} \right),$$

where $X_{i,m}$ is the position of the i-th male; $X_{best,f}$ is the best position in female snake group. $rand$ is a random number [0,1]; FM is the male fighting force.

② Mating pattern

15 $$X_{i,m} \left( {t + 1} \right) = X_{i,m} \left( t \right) + c_{3} \times M_{m} \times rand \times \left( {Q \times X_{i,f} - X_{i,m} \left( t \right)} \right),$$

16 $$X_{i,f} \left( {t + 1} \right) = X_{i,f} \left( t \right) + c_{3} \times M_{f} \times rand \times \left( {Q \times X_{i,m} - X_{i,f} \left( t \right)} \right),$$

where $X_{i,m}$ is the position of the i-th male; $X_{i,f}$ is the position of the i-th female; $rand$ is a random number [0,1]. $M_{m}$ and $M_{f}$ represent the mating ability of males and females, respectively.

**Figure 1:**
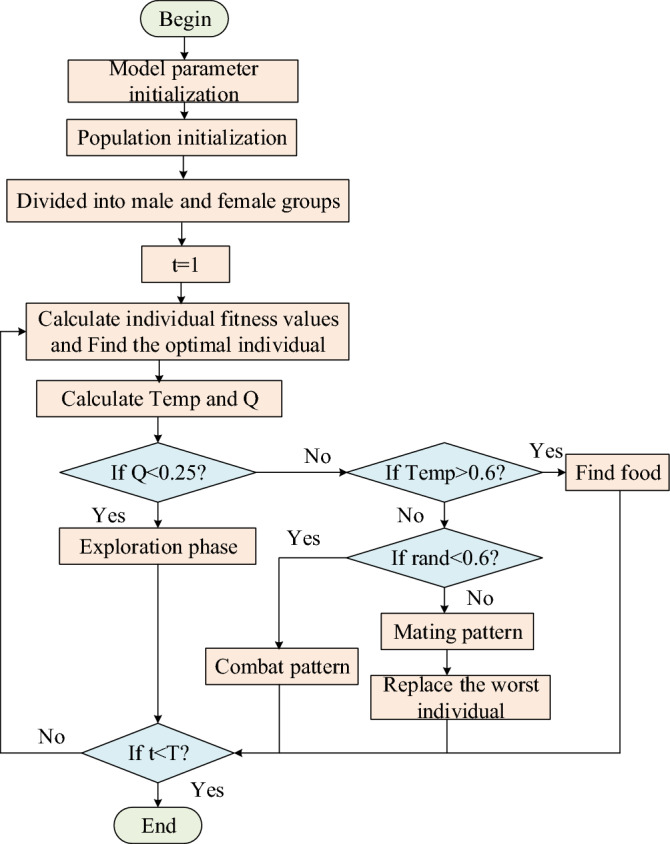
SO algorithm flow chart.

The specific implementation flow of SO algorithm is shown in Fig. 1.

### Kernel principle component analysis

KPCA^33^ is a method that transforms defective sample data into a high-dimensional space using a kernel function, then acquires essential low-dimensional data features within a linear subspace. This approach both maximizes the preservation of critical fault information and removes correlations among fault features. The specific steps can be described as follows:

Mapping the faulty dataset to a high-dimensional space $\Phi$, forming a new dataset $\Phi \left( {e_{i} } \right) = \left\{ {\Phi \left( {e_{1} } \right),\Phi \left( {e_{2} } \right), \ldots ,\Phi \left( {e_{n} } \right)} \right\},i = 1,2, \ldots ,n$. Assuming the samples in the high-dimensional space are already centered, the covariance matrix is as shown in Eq. (17):

17 $$C = \frac{1}{n}\sum\limits_{i = 1}^{n} {\Phi \left( {e_{i} } \right)} \Phi \left( {e_{j} } \right)^{T} = \frac{1}{n}\Phi \Phi^{T} .$$

Introducing the kernel function $K^{*} \eta = \Phi^{T} \Phi$, perform feature decomposition on the data in C, as shown in Eq. (18):

18 $$K^{*} \eta = \lambda \eta ,$$

where $\lambda$ represents the eigenvalues, and $\eta$ represents the eigenvectors.

Setting the cumulative contribution rate to 85%, arrange them in descending order and select the top c eigenvalues $\lambda_{j} \left( {j = 1,2, \ldots ,c} \right)$ along with their corresponding eigenvectors $\eta_{j} \left( {j = 1,2, \ldots ,c} \right)$, as specified in Eq. (19):

19 $$\frac{{\sum\limits_{j = 1}^{s} {\lambda_{j} } }}{{\sum\limits_{i = 1}^{s} {\lambda_{i} } }} \ge 85\% .$$

When the cumulative contribution rate reaches the specified requirement, calculate the nonlinear samples G after dimensionality reduction mapping, as specified in Eq. (20):

20 $$G = \left[ {\sum\limits_{j = 1}^{n} {\eta_{i} \Phi \left( {e_{i} } \right)^{T} } } \right] = \eta^{T} \left[ {\Phi \left( {e_{1} ,e} \right),\Phi \left( {e_{2} ,e} \right), \ldots ,\Phi \left( {e_{i} ,e} \right)} \right]^{T} .$$

### Fault diagnosis flow of transformer under unbalanced small sample condition

**Figure 2:**
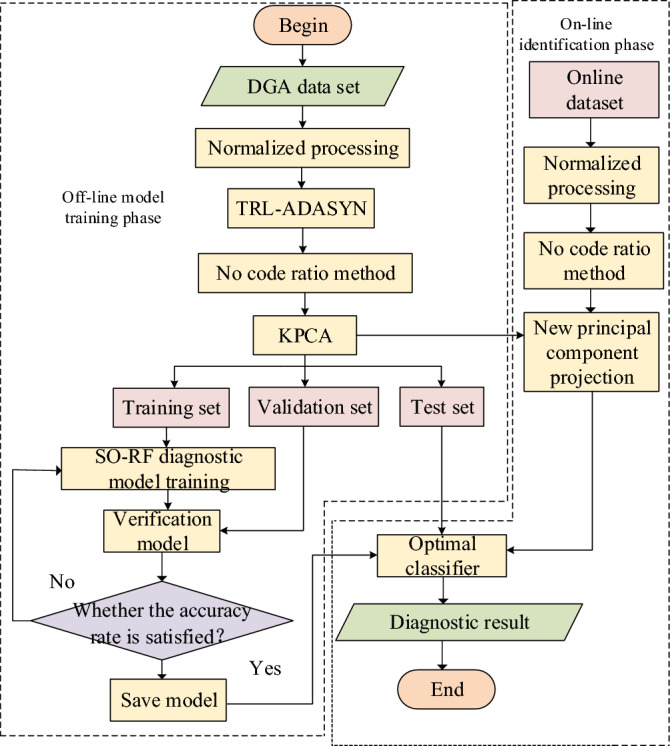
Fault diagnosis flow chart.

In this paper, an effective transformer fault diagnosis method is proposed from three perspectives: category unbalance processing, feature extraction and pattern recognition. The specific flow chart is shown in Fig. 2, which mainly includes two stages: offline model training and online recognition.

The off-line model training stage is mainly divided into the following four steps.

*Step 1* Standardize the collected DGA sample data, use TLR to remove the boundary data and noise of the training set, and then use ADASYN to expand the data of a few classes of samples.

*Step 2* The 18-dimensional feature is constructed by using the code-free ratio method, and the feature fusion is carried out by KPCA to remove the redundant information, and then divided into the training set, verification set and test set according to the proportion.

*Step 3* Optimize the parameters of n_estimators and max_depth of decision tree in RF model by SO algorithm.

*Step 4* Verify the accuracy of each iteration model with verification set. When the accuracy is improved less than 0.001 after two consecutive trainings, complete the model training and save the model parameters; otherwise, re-train the model until the conditions are met. Then the test set is sent into the trained SO-RF model to check the diagnostic accuracy of the model.

The online identification stage is mainly divided into the following three steps.

*Step 1* Normalize the transformer fault samples collected in real time.

*Step 2* The 18-dimensional feature is constructed using the uncoded ratio method, and then the fusion feature is obtained by projecting to the best principal element.

*Step 3* Feed the fusion features into the optimal classification model to identify the transformer state.

### Model evaluation index

In traditional transformer fault diagnosis, the commonly used diagnostic metric is the accuracy rate, which is a single measure and doesn’t effectively distinguish between misclassifications and missed detections. To address this limitation, this paper introduces several comprehensive accuracy metrics for transformer fault diagnosis, including the recall ratio (R), precision ratio (P), Kappa coefficient, and F1 index. The recall ratio (R) represents the rate of missed detections for a specific fault type, while the precision ratio (P) represents the rate of misclassifications for a specific fault type. In practical scenarios, the recall rate may be high while the accuracy rate is low, or vice versa. To balance both aspects, the F1 index is introduced. The F1 index is a measure of the harmonic average between the recall rate and precision rate. A higher F1 value indicates better model performance. The specific formula is as follows:

21 $${\text{R}} = TP/\left( {TP + FN} \right),$$

22 $${\text{P}} = TP/\left( {TP + FP} \right),$$

23 $$F1 = 2PR/\left( {P + R} \right),$$

where TP indicates that the fault sample is determined. And determine the correct number; FP represents the number of normal sample decisions made, but the decision is wrong; FN indicates the number of normal sample decisions made, but the decision is wrong.

The Kappa coefficient formula is as follows:

24 $$k = {{\left( {P_{0} - P_{e} } \right)} \mathord{\left/ {\vphantom {{\left( {P_{0} - P_{e} } \right)} {\left( {1 - P_{e} } \right)}}} \right. \kern-0pt} {\left( {1 - P_{e} } \right)}},$$

where P_0_ is the sum of the number of correctly classified samples of each class divided by the total number of samples; Pe is the sum of the product of the actual and predicted quantities for all categories, divided by the square of the total number of samples. Generally, the results of Kappa calculation fall between [0,1] and can be divided into five groups to represent different levels of consistency, namely: very low consistency, general consistency, medium consistency, high consistency and almost complete consistency. When used as an evaluation index of the model, the closer the calculated value is to 1, the better the diagnostic effect of the model is.

## Example analysis

In this paper, 338 sets of monitoring data provided by a power supply company in Zhejiang, China, were selected as a sample set, including 7 different operating states of medium discharge and overheat, low temperature overheat, high temperature overheat, partial discharge, low energy discharge, high energy discharge and normal, which were respectively represented by labels 1–7. Each operating state includes five characteristic gases, H_2_, CH_4_, C_2_H_4_, C_2_H_6_ and C_2_H_2_. The number of samples for each category is shown in Table 1.

**Table 1:** Category label and sample distribution.

Transformer status type	Sample size	Class tag
Normal	151	1
High temperature superheating	64	2
Medium and low temperature superheating	36	3
High energy discharge	32	4
Low energy discharge	25	5
Partial discharge	20	6
Discharge and superheat	10	7

### Transformer fault data preprocessing and feature selection

When the transformer fails, the composition and concentration of dissolved gas in the insulation oil will change. Therefore, the content of dissolved H_2_, CH_4_, C_2_H_4_, C_2_H_6_ and C_2_H_2_ in the transformer oil is used as the basis for transformer fault diagnosis. The content of each gas is normalized, as shown in formula (25):

25 $$x_{i}^{*} = \frac{{x_{i} - x_{i\,\,\min } }}{{x_{i\,\,\max } - x_{i\,\,\min } }},$$

where $x_{i}$ and $x_{{\text{i}}}^{*}$ are the characteristics before and after normalization; ${\text{x}}_{{{\text{i}}\max }}$ and ${\text{x}}_{{{\text{i}}\min }}$ represents the original minimum and maximum values before normalization. In order to deeply explore the correlation between the ratio of dissolved gas content in oil and the fault type, the 18-dimensional joint feature is constructed by using the non-coding ratio method. Where, THC = CH_4_ + C_2_H_4_ + C_2_H_6_ + C_2_H_2_, ALL = H_2_ + CH_4_ + C_2_H_4_ + C_2_H_6_ + C_2_H_2_, as shown in Table 2.

**Table 2:** Characteristic coding and characteristic quantity of dissolved gas in oil.

Feature coding	Characteristic quantity	Feature coding	Characteristic quantity
1	CH_4_/H_2_	10	C_2_H_4_/THC
2	C_2_H_2_/H_2_	11	C_2_H_6_/THC
3	C_2_H_2_/C_2_H_4_	12	C_2_H_2_/THC
4	C_2_H_4_/C_2_H_6_	13	(CH_4_ + C_2_H_4_)/THC
5	C_2_H_6_/CH_4_	14	H_2_/ALL
6	C_2_H_2_/CH_4_	15	CH_4_/ALL
7	C_2_H_4_/CH_4_	16	C_2_H_2_/ALL
8	H_2_/THC	17	C_2_H_4_/ALL
9	CH_4_/THC	18	C_2_H_6_/ALL

### Data balancing processing

As indicated in Table 1, normal samples constituted 45.07% of the total samples, while partial discharge, low-energy discharge, and discharging-over-heat samples represented 7.40%, 5.92%, and 2.99% of the total samples, respectively. Such data imbalance could lead to the misclassification of a few samples as normal, resulting in diminished recognition accuracy. To address this issue, this paper employs the TLR algorithm to filter out noise and boundary data from the training set. Subsequently, the ADASYN algorithm is utilized to augment the number of fault samples. The distribution of sample quantities before and after this processing is presented in Table 3.

**Table 3:** Comparison before and after fault sample preprocessing.

Transformer status type	Number of raw training data	Amount of data after balanced processing
Normal	151	151
High temperature superheating	64	151
Medium and low temperature superheating	36	151
High energy discharge	32	151
Low energy discharge	25	151
Partial discharge	20	151
Discharge and superheat	10	151

### Feature selection

**Figure 3:**
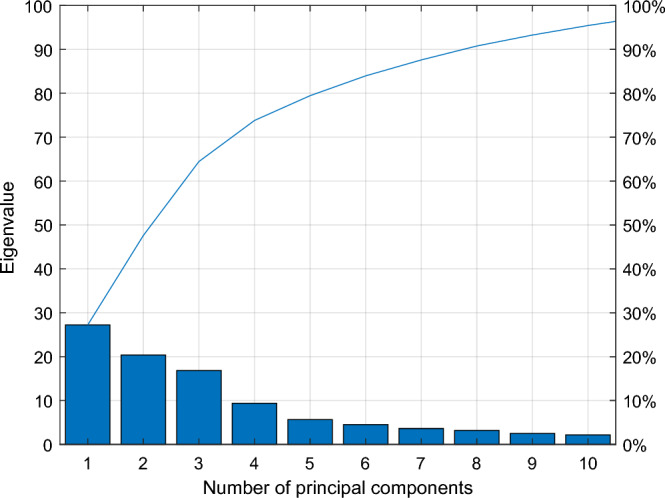
Cumulative contribution rate.

To mitigate the inclusion of redundant information in fault features, Kernel Principal Component Analysis (KPCA) was utilized to integrate the constructed 18-dimensional joint features. The contribution rates and cumulative contribution rates of each principal component are visualized in Fig. 3. Within this figure, it is evident that the initial principal component encompasses the majority of feature information, and as the number of principal components increases, the volume of feature information decreases. The cumulative contribution rate associated with each principal component was calculated as per Formula (19) and is presented in Table 4.

**Table 4:** Cumulative contribution rates of variance for each principal components.

The number of principal components	1	2	3	4	5	6
Variance values	27.2308	20.3735	16.8472	9.3583	5.6418	4.5176
Cumulative contribution rate (%)	27.2308	47.6043	64.4515	73.8098	79.4516	83.9692
The number of principal components	**7**	8	9	10	11	12
Variance values	3.6268	3.1760	2.4942	2.1714	1.8969	1.0946
Cumulative contribution rate (%)	87.596	90.7736	93.2678	95.4392	97.3361	98.4307
The number of principal components	13	14	15	16	17	18
Variance values	0.6905	0.4741	0.2336	0.1201	0.0440	0.0007
Cumulative contribution rate	99.1212	99.5953	99.8289	99.4940	99.9930	100

**Figure 4:**
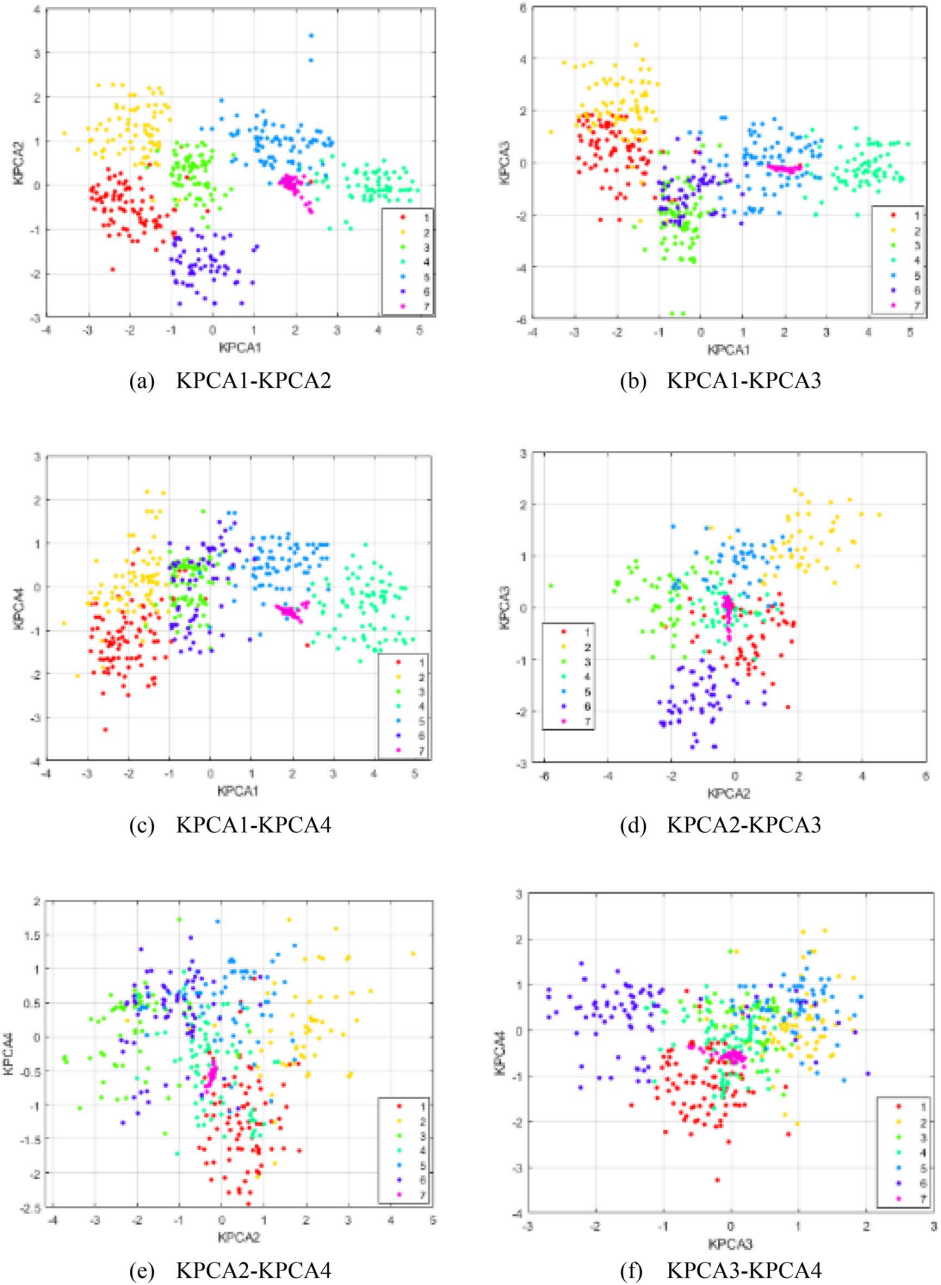
Scatter plot of different principal elements.

As illustrated in Table 4, the cumulative variance contribution rate of the first seven principal components reaches 0.876. This signifies that these initial seven principal components capture over 85% of the explanatory power inherent in all principal components. Consequently, the first seven principal components are chosen as the inputs for the transformer fault diagnosis model. To further underscore the efficacy of KPCA feature fusion, two-dimensional scatter plots are generated for distinct principal components, as visualized in Fig. 4. The scatter plot in Fig. 4 reveals that the clustering effect is most pronounced in the first and second principal components, with the clustering effect diminishing progressively for subsequent principal components.

### Fault diagnosis result

Fusion features extracted from KPCA were divided into training set, test set and verification set according to the ratio of 6:2:2, as shown in Table 5.

**Table 5:** Distribution of sample data.

Transformer status type	Training set	Validation set	Test set
Normal	93	29	29
High temperature superheating	93	29	29
Medium and low temperature superheating	93	29	29
High energy discharge	93	29	29
Low energy discharge	93	29	29
Partial discharge	93	29	29
Discharge and superheat	91	30	30

**Figure 5:**
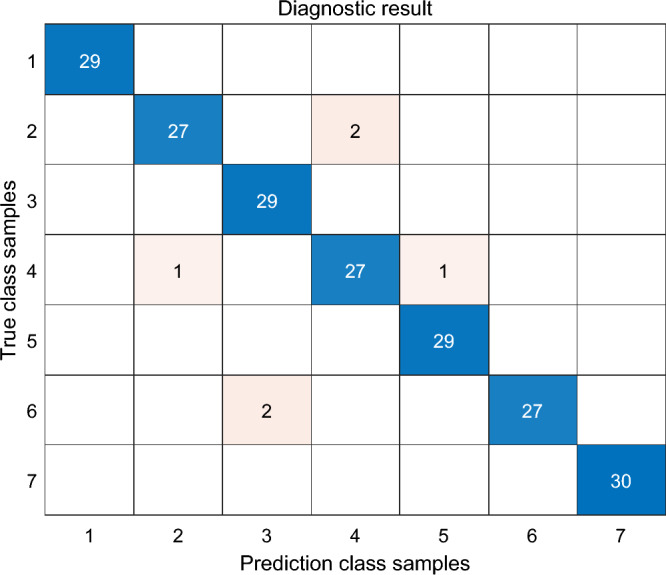
Confusion matrix of fault diagnosis classification.

To obtain the optimal diagnostic model, the SO algorithm was employed to optimize the n_estimators and max_depth of decision trees within the RF model. A population size of 30 and a maximum iteration count of 100 were set. The search range for the number of decision trees was (0, 100), and the search range for decision tree depth was (0, 20). The simulations in this study were conducted using MATLAB 2018b software, and the resulting confusion matrix is shown in Fig. 5. From Fig. 5, it can be observed that out of the 204 samples in the test set, 198 were correctly diagnosed, resulting in an overall accuracy of 97.06%. Specifically, the accuracy of diagnosing medium and low-temperature overheating, partial discharge, and combined discharge and overheating faults was 100%. Based on the data in the confusion matrix, the diagnostic model’s precision (P), recall (R), and F1-score were calculated as 0.9704, 0.9711, and 0.9707, respectively. Additionally, the Kappa coefficient of the diagnostic model was 0.9659, indicating almost perfect agreement, further confirming the high fault recognition accuracy and excellent stability of the model proposed in this study.

## Results and discussion

### Qualitative and quantitative analysis of TLR-ADASYN data equalization

**Figure 6:**
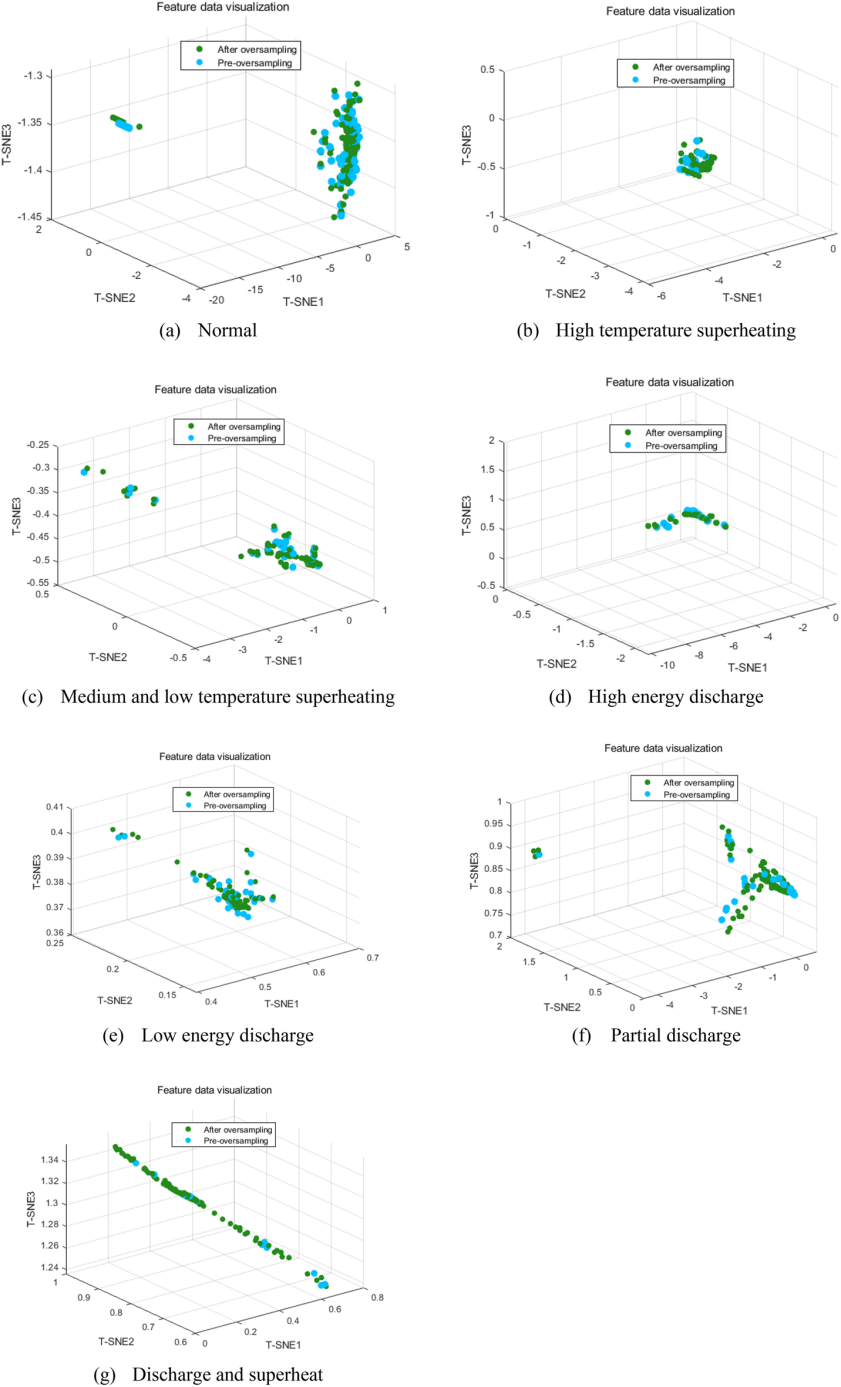
Data distribution trend of different types of faults before and after balanced processing.

To validate the effectiveness of the TRL-ADASYN sampling method, this study conducts a comprehensive performance comparison of various sampling methods, combining qualitative observations with quantitative analysis. Firstly, to visually demonstrate that the TRL-ADASYN sampling method successfully augments the sample size while preserving essential data characteristics, the study employs t-distributed Stochastic Neighbor Embedding (t-SNE)^34^ to map transformer dissolved gas data into a three-dimensional space for visualization, as depicted in Fig. 6. In Fig. 6, the blue dots represent samples after applying the sampling method, while the orange dots represent samples before sampling. Within this three-dimensional coordinate graph, it becomes evident that the data distribution patterns of different fault types remain consistent both before and after the implementation of the TRL-ADASYN sampling method. Furthermore, the statistical characteristics align, providing compelling evidence for the validity and reliability of the augmented data.

Secondly, we conducted a quantitative comparison of the performance of various sampling methods, evaluating five different treatment approaches, namely, non-equilibrium dataset, random oversampling, SMOTE oversampling, ADASYN oversampling, and ROS downsampling. The resulting diagnostic outcomes are presented in Table 6. As illustrated in Table 6, the diagnostic accuracy of the original dataset, without undergoing any balancing processing, stood at 88.24%, accompanied by a Kappa coefficient of 0.8654. The adoption of oversampling or downsampling algorithms led to varying degrees of improvement in diagnostic accuracy. However, when the downsampling algorithm was employed, valuable information was lost due to the removal of a portion of the majority class sample data. Comparatively, in contrast to ADASYN, SMOTE, and random oversampling, the diagnostic accuracy of the method proposed in this paper increased by 0.59%, 1.96%, and 4.41%, respectively. Furthermore, the Kappa coefficient also witnessed an increase of 0.0057, 0.0224, and 0.0505, respectively. The experimental results conclusively demonstrate that the approach introduced in this paper effectively addresses the issue of insufficient sample distribution in certain classes, mitigating the potential decline in diagnostic accuracy caused by a model's inclination toward the majority class samples.

**Table 6:** Diagnostic results under different sampling methods.

Data enhancement method	Accuracy rate (%)	Kappa coefficient
TRL-ADASYN	97.06	0.9659
ADASYN	95.59	0.9489
SMOTE	94.12	0.9322
Random oversampling	91.67	0.9041
ROS downsampling	88.24	0.8654
Unbalanced data sets	78.57	0.7164

### Comparative analysis of diagnostic results under different characteristics

**Figure 7:**
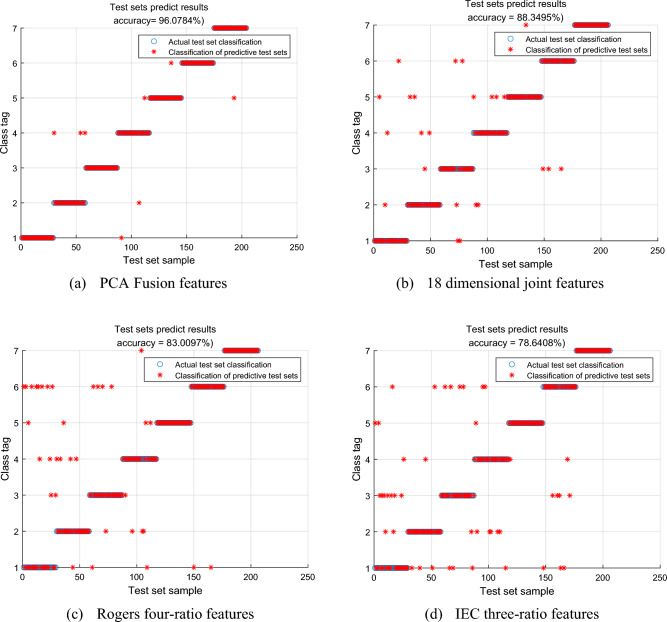
Comparison of diagnostic results of different feature inputs.

The use of KPCA feature extraction also has a significant impact on improving diagnostic accuracy. In this study, oversampled IEC three-ratio features, Rogers’ four-ratio features, 18-dimensional joint features, and the first 7 dimensions of features extracted using principal component analysis were analyzed and compared, as shown in Fig. 7. In the figure, the red dots represent samples in the test set that were correctly classified, while the blue circles represent samples with their true classifications. The scattered points indicate samples misclassified as other categories, and a higher number of scattered sample points indicates lower diagnostic accuracy. From Fig. 7, it can be observed that the use of IEC three-ratio features and Rogers’ four-ratio features have more scattered points compared to the 18-dimensional joint features, indicating that the 18-dimensional joint features are better at exploring the relationship between fault types and dissolved gases in the oil. Table 7 shows that the corresponding Kappa coefficients for the four different features are 0.9433, 0.9209, 0.8821, and 0.8543. Using KPCA fusion features reduced the feature dimensionality, significantly improving fault diagnosis accuracy, thus confirming the superiority of this method.

**Table 7:** Comparison of Kappa coefficients of different characteristics.

Features name	KPCA Fusion feature	PCA Fusion features	18 dimensional joint features	Rogers four ratio features	IEC ratio features
Kappa coefficient	0.9659	0.9567	0.8587	0.8824	0.7592

### Comparative analysis of different fault diagnosis models

To illustrate the effectiveness of this diagnosis method, comparison and analysis were made with GA-XGBoost diagnosis model proposed in Ref.^35^, PSO-BiLSTM diagnosis model proposed in Ref.^36^ and WOA-SVM diagnosis model proposed in Ref.^37^, and the diagnostic results were shown in Table 8. It shows the superiority of the diagnostic model proposed in this paper.

**Table 8:** Comparison of diagnostic results of different models.

Model name	GA-XGBoost	PSO-BiLSTM	WOA-SVM	SO-RF
Diagnostic accuracy	92.70%	92.50	91.11%	96.08%

**Figure 8:**
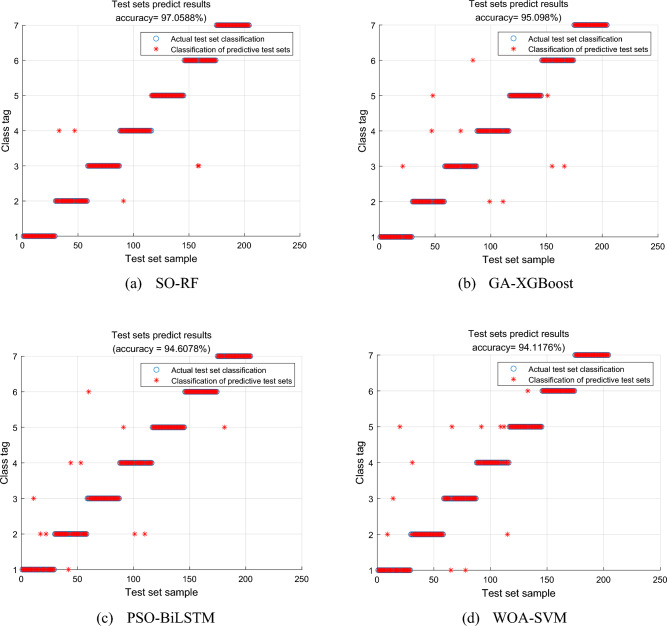
Comparison of results of different diagnostic models.

The 7-dimensional fused and dimensionally reduced features were separately input into three different models, GA-XGBoost, PSO-BiLSTM, and WOA-SVM, for comparative analysis against the diagnostic model proposed in this study. The diagnostic results are shown in Fig. 8, and the model evaluation metrics are compared in Table 9. From the information presented in the figure and the table, it can be observed that the SO-RF model had the fewest misclassified samples, resulting in an accuracy improvement of 1.47%, 2.45%, and 3.43% compared to the GA-XGBoost, PSO-BiLSTM, and WOA-SVM diagnostic models, respectively. In comparison with the recognition accuracy in the original literature, the improvement was 1.91%, 1.13%, and 1.54%, respectively. Furthermore, in terms of evaluation metrics such as recall, precision, and F1 score, the method proposed in this study exhibits more stable performance compared to other models. From the perspective of the Kappa coefficient, the method presented in this study achieved a score of 0.9546, indicating almost perfect agreement. This further underscores the effectiveness of the feature extraction method and fault diagnostic model proposed in this study.

**Table 9:** Comparison of model evaluation indexes.

Diagnostic model name	GA-XGBoost	PSO-BiLSTM	WOA-SVM	SO-RF
Recall ratio	0.9507	0.9460	0.9409	0.9704
Precision ratio	0.9515	0.9466	0.9431	0.9711
F1 value	0.9511	0.9463	0.9420	0.9707
Kappa coefficient	0.9620	0.9377	0.9321	0.9659
Diagnostic accuracy	95.10%	94.61%	94.12%	97.06%

### The generalization performance analysis of the model

Additional datasets were employed to assess the model’s ability to generalize. Specifically, the IEC TC 10^38^ public dataset was selected for this purpose. In accordance with the categorization provided in Ref. ^39^, transformer fault types were classified into six categories: medium and low-temperature overheating, high-temperature overheating, low energy discharge, high energy discharge, partial discharge, and normal operation, denoted as labels 1 to 6, respectively. Leveraging the diagnostic techniques proposed in this study, the diagnostic outcomes are presented in Table 10.

**Table 10:** Diagnostic results under the IEC TC 10 data set.

Data set name	Diagnostic accuracy	Kappa coefficient
IEC TC 10	93.98%	0.9276

As depicted in Table 10, the diagnostic accuracy for the IEC TC 10 dataset stands at 93.98%, accompanied by a Kappa coefficient of 0.9276. This underscores the robust generalization capabilities of the approach introduced in this paper when compared to the previously cited model.

## Conclusion

Aiming at the problem of misjudgment and missing judgment of a few types of samples caused by unbalanced transformer fault samples, a transformer fault diagnosis method under the condition of unbalanced small samples is proposed, and the following conclusions are drawn through practical data simulation:

1. The TLR-ADASYN method adopted in this paper can effectively solve the problem of low diagnostic accuracy caused by insufficient and unbalanced transformer fault sample data. In addition, the use of KPCA for feature fusion can avoid the appearance of redundant information and further improve the accuracy of the model.
2. Compared with GA-XGBoost, PSO-BiLSTM and WOA-SVM diagnostic models, the accuracy of SO-RF model proposed in this paper reached 96.08%, and the Kappa coefficient reached 0.9546, which were superior to other models. The results show that SO-RF model has better stability and generalization.

However, using dissolved gases in oil as an early diagnostic method for transformers, relying solely on these gases as input features is insufficient to reflect the overall condition of the transformer. Therefore, future work can collect vibration signal data as additional input for the model. Furthermore, the diagnostic model proposed in this paper did not take into account external factors and the influence of the transformer's inherent characteristics on fault diagnosis accuracy. Subsequent research should consider the impact of external factors on the fault diagnosis model.

## Data Availability

The datasets generated and/or analysed during the current study are not publicly available due [The data set is a company secret] but are available from the corresponding author on reasonable request. E-mail: xhaiqi0526@163.com.
